# Transmission of *Bartonella henselae* by *Ixodes ricinus *

**DOI:** 10.3201/eid1407.071110

**Published:** 2008-07

**Authors:** Violaine Cotté, Sarah Bonnet, Danielle Le Rhun, Evelyne Le Naour, Alain Chauvin, Henri-Jean Boulouis, Benoit Lecuelle, Thomas Lilin, Muriel Vayssier-Taussat

**Affiliations:** * Institut National de la Recherche Agronomique, Maisons-Alfort, France; † École Nationale Vétérinaire de Nantes, Nantes, France; ‡ École Nationale Vétérinaire d’Alfort, Maisons-Alfort, France

**Keywords:** Tick-borne pathogen, Bartonella henselae, Ixodes ricinus, transmission, research

## Abstract

*Bartonella* spp. are facultative intracellular bacteria associated with several emerging diseases in humans and animals. *B*. *henselae* causes cat-scratch disease and is increasingly associated with several other syndromes, particularly ocular infections and endocarditis. Cats are the main reservoir for *B. henselae* and the bacteria are transmitted to cats by cat fleas. However, new potential vectors are suspected of transmitting *B. henselae*, in particular, *Ixodes ricinus*, the most abundant ixodid tick that bites humans in western Europe. We used a membrane-feeding technique to infect *I. ricinus* with *B. henselae* and demonstrate transmission of *B. henselae* within *I. ricinus* across developmental stages, migration or multiplication of *B. henselae* in salivary glands after a second meal, and transmission of viable and infective *B. henselae* from ticks to blood. These results provide evidence that *I. ricinus* is a competent vector for *B. henselae*.

## Abstract 2

The causative agent of cat-scratch disease in humans can be transmitted by this tick through saliva.

*Bartonella* spp. are facultative intracellular bacteria associated with several emerging diseases in humans and animals (*1*). Domestic animals and wildlife represent a large reservoir for *Bartonella* spp., and at least 10 species or subspecies have been reported to cause zoonotic infections. *B*. *henselae* causes cat-scratch disease, possibly the most common zoonosis acquired from domestic animals in industrialized countries and is becoming increasingly associated with other syndromes, particularly ocular infections and endocarditis (*2*–*6*). Although cat fleas are well-established vectors for *B*. *henselae* (*7*–*10*), transmission by other arthropods, in particular ticks, has been suggested (*11*–*13*). *Ixodes ricinus* is the most widespread and abundant ixodid tick in western Europe and is frequently associated with bites in humans. It is a vector of emerging zoonotic pathogens including *Borrelia burgdorferi* sensu lato (*14*), *Anaplasma phagocytophilum* (*15*), and *Babesia* spp (*16*).

Direct proof of transmission of *Bartonella* spp. by a tick was reported by Noguchi in 1926 (*17*), who described experimental transmission of *B*. *bacilliformis* (cause of Oroya fever) to monkeys by *Dermacentor andersoni*. In this study, ticks were allowed to feed on infected monkeys for 5 days. After removal, partially engorged ticks were placed on healthy monkeys in which disease then developed. This study showed that ticks could acquire and transmit the bacteria but did not demonstrate their vector competence or transtadial transmission throughout the tick’s life cycle. Since this early study, the role of ticks in *Bartonella* spp. transmission has been strongly implied but never definitively demonstrated. *Bartonella* spp. DNA was detected in questing and engorged nymphs and adults *Ixodes* spp. collected in North America, Europe, and Asia (*13*,*18*–*26*). If one considers that ixodid ticks feed only once per stage, *Bartonella* spp. DNA in questing ticks suggests transtadial transmission of these bacteria.

Other observations support *Bartonella* spp. transmission by ticks. Co-occurrence of *Bartonella* spp. with known tick-borne pathogens such as *B*. *burgdorferi* sensu lato, *A*. *phagocytophilum*, or *Babesia* spp. is not a rare event in ticks and hosts (*13*,*19*,*24*,*27*). A study conducted in a veterinary hospital in the United States (California) demonstrated that all dogs with endocarditis and infected with *Bartonella* spp. were also seropositive for *A*. *phagocytophilum* (*28*). In humans, several case studies have reported patients with concurrent *Bartonella* seropositivity and detection of *Bartonella* spp. DNA in their blood, along with *B*. *burgdorferi* infection of the central nervous system after tick bites (*11*,*29*). Moreover, *Bartonella* spp. DNA has been detected in human blood cells after a tick bite (*30*), and 3 patients with *B*. *henselae* bacteremia who had no history of contact with cats but had sustained tick bites were reported in Texas (*12*). Finally, tick exposure was determined to be a risk factor associated with *B*. *vinsonii* seropositivity in dogs (*31*).

Because *Bartonella* spp. are emerging human pathogens and *Ixodes* spp. can transmit a large spectrum of pathogens to humans, the capability of *Ixodes* spp. in transmitting human pathogenic *Bartonella* spp. should be determined. We used a membrane-feeding technique to infect *I*. *ricinus* with *B*. *henselae*, and investigated transtadial and transovarial transmission of viable and infective bacteria and putative transmission from tick saliva to blood during artificial blood meals.

## Materials and Methods

### Ticks

*I*. *ricinus* ticks were collected from the forest of Gâvre (Loire-Atlantique, France) in 2006 by flagging vegetation as described (*32*). Ticks were reared and maintained in chambers with a relative humidity of 80%–90% at 22°C before feeding on artificial skin. A total of 217 whole ticks were tested for *Bartonella* DNA.

### Culturing of *B*. *henselae*

*B*. *henselae* (Houston-1 ATCC 49 882) were grown on 5% defibrinated sheep blood Columbia agar (CBA) plates, which were incubated at 35°C in an atmosphere of 5% CO_2_. After 10 days, bacteria were harvested, suspended in sterile phosphate-buffered saline (PBS), and used immediately for artificial feeding of ticks.

### Housing of Cats

Three healthy male European cats were used (12–14 months of age, weight = 3.2–4.6 kg at the beginning of the study). These cats, which were bred by Harlan (Indianapolis, IN, USA), were imported at 9 months of age. Before the study, animals underwent clinical examinations and showed no signs of disease. Absence of *Bartonella* spp. was confirmed by blood culture, tests for DNA of *Bartonella* spp., and serologic analysis. Animals were housed singly in cages in compliance with European guidelines (cage surface 6,000 cm^2^, cage height 50 cm). Animals were allowed to acclimate for 3 months to the facility, diet, and handling before the first blood sample was taken and subsequent infection. Animals were fed ad libitum with standard feline maintenance diet (Harlan) and received water with no restrictions. Animal care was provided in accordance with the good animal care practice.

### Feeding of *I. ricinus* Ticks with Ovine Blood

**Figure 1:**
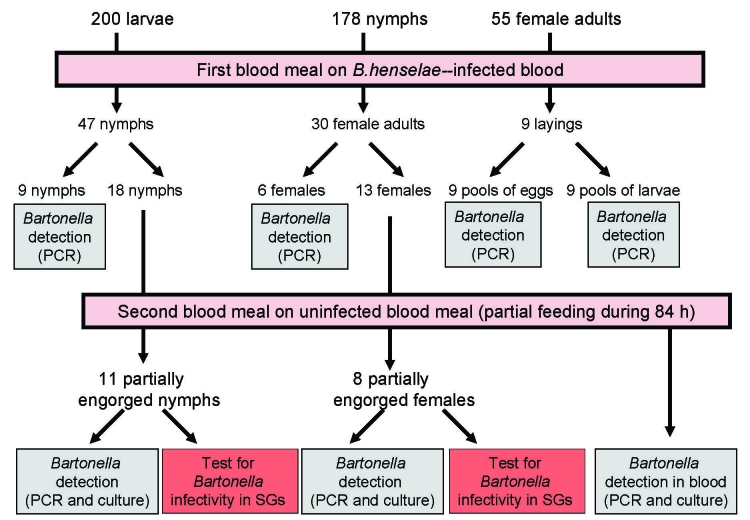
Experimental framework of *Ixodes ricinus* tick infection by *Bartonella henselae*–infected blood. Ticks (200 larvae, 178 nymphs, and 55 female adults) were engorged by feeding through artificial skin on *B. henselae*–infected blood for 5 days for larvae, 12 days for nymphs, and 21 days for adults. Larvae and nymphs were allowed to molt and engorged females were allowed to lay eggs. To evaluate transtadial and transovarial transmission, *Bartonella* spp. DNA was detected by PCR in salivary glands (SGs) and carcasses of 9 nymphs, 6 female adults, 9 pools of eggs, and resulting pools of larvae. Eighteen nymphs and 13 adult females fed on infected blood at preceding stages were refed for 84 h on noninfected blood. *Bartonella* spp. DNA was detected by PCR in SGs of 7 engorged nymphs and 3 engorged female adults. *B. henselae* colonies were isolated from SGs of 3 nymphs and 4 adults and from blood removed from feeders. Infectivity of *B. henselae* in SGs was tested by infecting 2 cats with 1 pair of SGs from a potentially infected nymph and 1 pair of SGs from a potentially infected adult, respectively.

The general experimental framework of artificial feeding is shown in Figure 1. Ovine blood used in all experiments was obtained from 3 sheep reared at the National Veterinary School in Nantes, France. Absence of *Bartonella* spp. in the blood of these sheep was confirmed by culture assay and tests for DNA of *Bartonella* spp. by PCR. Lithium heparin–coated vacutainer tubes (Becton Dickinson, Le Pont de Claix-Cedex, France) were used to draw blood by venipuncture. Blood was depleted of functional complement by heat treatment (incubation for 30 min at 56°C) before use. To avoid fungal and bacterial contamination during feeding experiments, decomplemented blood was supplemented with fosfomycin (20 μg/mL) and amphotericin B (0.25 μg/mL), which were previously determined to have no effect on *B*. *henselae* viability.

### Feeding of Ticks with *B. henselae*–Infected Blood

The method of artificial feeding used in this study was adapted from the method of Bonnet et al. (*33*). Ticks were placed in 75-cm^2^ tissue culture flasks pierced at the top to accommodate a 4-cm diameter glass feeder. The feeder apparatus was closed with a parafilm membrane at the top and a gerbil or rabbit skin membrane at the bottom. Gerbil skins were used for feeding larvae and rabbit skins for feeding nymphs and adults (*33*). To attract ticks, a constant temperature (37°C) was maintained by a water-jacket circulation system through the glass feeder. For blood infection, 10 μL of *B*. *henselae* suspension at a concentration of 10^9^ CFU/mL in PBS was added to 10 mL of blood supplemented with fosfomycin and amphotericin B. The culture box containing ticks was placed under the feeding apparatus, and 3 mL of *B*. *henselae*–infected ovine blood, changed twice a day, was introduced until the ticks were replete. To feed adult ticks, an equal number of males (for reproduction) and females were used. Separate apparatuses were used to engorge 200 larvae, 178 nymphs, and 55 female adults. As a control, 50 nymphs were fed under the same conditions on uninfected decomplemented ovine blood.

After feeding, larvae and nymphs were allowed to molt to nymphs and adults, respectively, and engorged females were allowed to lay eggs. Between each feeding on skin, ticks were starved for at least 2 months.

### Feeding with Uninfected Blood of Ticks Infected with *B. henselae* at Preceding Stages

Nymphs and adults fed on blood infected with *B*. *henselae* at preceding stages were fed with uninfected, decomplemented, ovine blood in 2 glass feeders as described above. After 84 h of refeeding, nymphs and females attached to the skin were removed and dissected. *Bartonella* spp. DNA was detected every 24 h in blood from the first 48 h of attachment onward. At each time point, 3 mL of blood was removed and centrifuged for 30 min at 3,000 × *g*. The supernatant was aspirated, and the pellet (200 μL) was used to detect bacterial DNA. After 84 h of feeding, 10 μL of blood was used for *B*. *henselae* culture.

### Tick Dissection

Salivary glands (SGs) from *I*. *ricinus* adults and nymphs were dissected under a magnifying glass in sterile PBS. All dissection material was cleaned with DNA-off (Eurobio, Courtaboeuf, France) and rinsed with sterile water between each sample. Individual pairs of tick SGs and the remaining tick carcasses were suspended in 150 μL of PBS before culture and used to infect cats or frozen at –80°C until DNA extraction.

### Detection of *B*. *henselae* in Tick and Blood Samples

#### DNA Extraction

Carcasses (ticks without SGs), entire ticks, and pools of 50 larvae were mechanically disrupted as described (*34*). Eggs were crushed by using a microtissue grinder and dissected SGs were directly used for DNA extraction. DNA was extracted from all tick samples by using the Nucleospin Tissue kit according to the manufacturer’s instructions (Macherey-Nagel, Duren, Germany). DNA was extracted from blood samples by using the Nucleospin Blood Quick Pure kit (Macherey-Nagel). For carcasses and entire ticks, the final elution volume was 100 μL for adults and 30 μL for nymphs. For DNA from SGs, the final elution volume was 50 μL for adults and 20 μL for nymphs. Pools of eggs, larvae, and blood DNA were eluted in volumes of 50 μL.

#### PCR Amplification

Efficiency of tick DNA extraction was evaluated in all samples by amplification of a fragment of the tick mitochondrial 16S rRNA gene by using tick-specific primers TQ16S+1F (5′-CTGCTCAATGATTTTTTAAATTGCTGTGG-3′) and TQ16S-2R (5′-ACGCTGTTATCCCTAGAG-3′) as described (*35*). A seminested PCR was used to detect *B*. *henselae* DNA. Amplification was initially performed with 5 μL of DNA extract and universal bacteria primers amplifying a 535-bp fragment of the 16S rRNA gene: pc535 (5′-GTATTACCGCGGCTGCTGGCA-3′) and p8 (5′AGAGTTTGATCCTGGCTCAG-3′).

The second seminested amplification was performed with 5 μL of a 100-fold dilution of the first PCR product and primers pc535 and bsp16F (5′-TCTCTACGGAATAACACAGA-3′) a 16S rRNA *Bartonella* spp.–specific primer, and resulted in amplification of a 337-bp product. The PCR cycle was identical for both amplification reactions: an initial denaturation step for 8 min at 94°C; 35 cycles of denaturation for 1 min at 94°C, annealing for 1 min at 54°C, and extension for 1 min at 72°C; and a final extension step at 72°C for 10 min. Each reaction was conducted in a total volume of 25 μL with 0.5 μmol/μL of each primer, 2.5 mmol/L of each dNTP, 2.5 μL of 10× PCR buffer, and 1 U of *Taq* DNA polymerase (Takara Biomedical Group, Shiga, Japan). Negative (ticks fed on uninfected ovine blood) and positive (*B*. *bacilliformis* DNA to easily detect any cross-contamination) controls were included in each assay. All PCRs were performed in a thermocycler MyCycler (Bio-Rad, Strasbourg, France).

### Sequencing and Sequence Analysis

The expected 337-bp PCR product was isolated by agarose gel electrophoresis, excised from the gel, purified by using NucleoSpin Extract II (Macherey-Nagel), and sent to QIAGEN (Hilden, Germany) for direct sequencing. Sequences were compared with known sequences listed in the GenBank nucleotide sequence databases by using the BLAST search option of the National Center for Biotechnology Information (www.ncbi.nlm.nih.gov/BLAST).

### *B. henselae* Culture from Tick SGs and Blood

After refeeding and dissection, 4 pairs of SGs from 4 potentially infected adults females and 3 nymphs were incubated in 1 mL of Schneider *Drosophila* medium (Invitrogen, Cergy-Pontoise, France) at 35°C in an atmosphere of 5% CO_2_. After 6 days of incubation, 10-μL samples were placed on CBA plates. Ten microliters of blood was removed from the glass feeder after 84 h of refeeding with adults suspected of having *B*. *henselae* and incubated for 6 days in Schneider *Drosophila* medium before being placed on CBA plates.

### Infection of Cats with Adult and Nymph SGs Potentially Infected with *B*. *henselae*

One cat was intravenously infected with 150 μL of PBS containing SGs from refed adult ticks suspected of having *B*. *henselae*; another cat was infected with 150 μL of PBS. Before handling, cats were anesthetized with ketamine (10–15 mg/kg bodyweight) and diazepam (100 μg/kg bodyweight) given intravenously. One milliliter of blood was obtained from the jugular vein at days 7, 14, and 21 postinfection, and 100 μL of blood dilutions (1:10, 1:100, and 1:1,000) was directly placed on sheep blood CBA plates. Colonies were counted and CFU/mL was estimated after incubation for 10 days at 35°C in an atmosphere of 5% CO_2_.

## Results

### Detection of *Bartonella* spp. DNA in Questing *I*. *ricinus* Ticks

We tested 217 ticks collected in the forest of Gavre to determine prevalence of *Bartonella* spp. DNA. None of the 98 nymphs, 49 female ticks, and 70 male ticks collected in the forest showed amplification of the *Bartonella* spp. 16S rRNA gene.

### Transstadial Transmission of *B*. *henselae* DNA by *I*. *ricinus* Ticks

A total of 433 *I*. *ricinus* ticks were used in 2 independent experiments with feeding on artificial skin. Of these ticks, 169 (84.5%) of 200 larvae, 111 (62.3%) of 178 nymphs, and 19 (34.5%) of 55 female adults were successfully engorged and spontaneously detached. After feeding and detachment, all ticks were maintained in humidity chambers to enable molting or laying of eggs. After 3 months, 47 larvae (27.8%) and 58 nymphs (30.1%) molted into nymphs and adults (28 males and 30 females), respectively. Of the 19 engorged females, 9 laid eggs.

**Figure 2:**
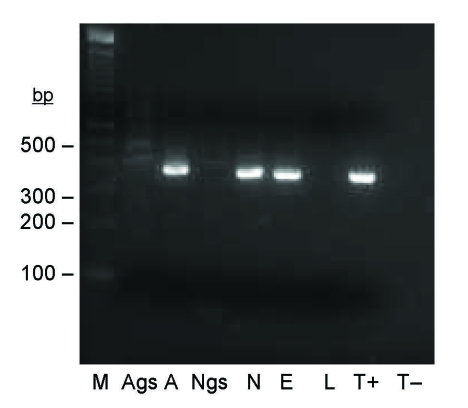
Seminested PCR detection of *Bartonella* spp. DNA in *Ixodes ricinus* ticks fed on *B. henselae*–infected ovine blood at preceding stage. Lane M, 100-bp DNA molecular mass marker; lane Ags, salivary glands of a female adult fed on infected blood as a nymph; lane A, carcass of a female adult fed on infected blood as a nymph; lane Ngs, salivary glands of a nymph fed on infected blood as a larva; lane N, carcass of a nymph fed on infected blood as a larva; lane E, eggs laid by female adult fed on infected blood; lane L, larvae hatched from female adult fed on infected blood; lane T+, *B. bacilliformis* DNA; lane T–, nymph fed on uninfected ovine blood.

Nine nymphs exposed to infection as larvae were tested for *B*. *henselae* DNA. All carcasses showed amplification of the expected 337-bp DNA fragment, and no amplification product was obtained from SGs. Four carcasses of 6 females exposed to *B*. *henselae* as nymphs showed the 337-bp DNA fragment, and no amplification product was obtained with SGs (Table, Figure 2). No amplified fragment was detected with DNA extracts from SGs or carcasses of control ticks fed on uninfected blood. All PCR products were sequenced and shared 100% identity with the 16S rRNA gene of *B*. *henselae* Houston-1 (Genbank accession no. BX897699).

**Table:** Transtadial transmission of *Bartonella* spp. DNA in *Ixodes ricinus* ticks after feeding through artificial skin on *B. henselae*–infected ovine blood

*I. ricinus* stage	PCR detection after infectious blood meal and molting, no. PCR-positive samples/no. samples tested (%)			PCR detection after partial refeeding, no. PCR-positive samples/no. samples tested (%)	
	Carcasses	Salivary glands		Carcasses	Salivary glands
Nymph	9/9 (100)	0/9 (0)		4/7 (57)	5/7 (71)
Female adult	4/6 (67)	0/6 (0)		1/3 (33)	2/3 (67)

### Transovarial Transmission of *B*. *henselae* DNA by *I*. *ricinus* Ticks

Among 9 pools of eggs laid by females fed on *B*. *henselae*–infected blood, 3 showed amplification of the expected *Bartonella* spp.–specific 337-bp DNA fragment. No amplification was obtained with larvae from eggs positive or negative for *Bartonella* spp. DNA (Figure 2).

### Transmission of Viable and Infective *B*. *henselae* by *I*. *ricinus* Ticks

**Figure 3:**
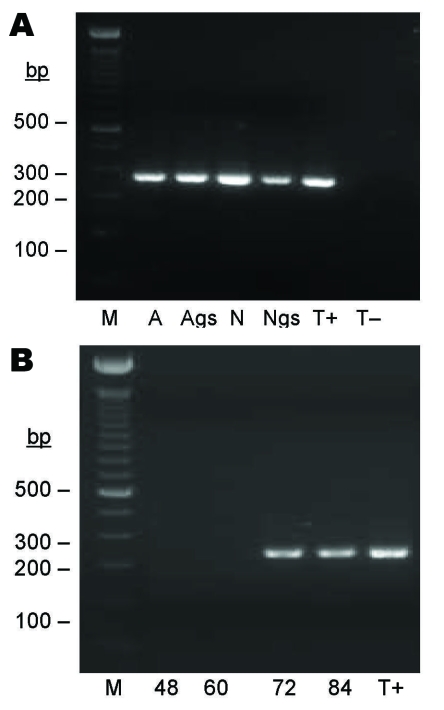
Seminested PCR detection of *Bartonella* spp. DNA after partial refeeding of infected ticks. A) *Bartonella* spp. DNA detection in *Ixodes ricinus* ticks fed on *B. henselae*–infected blood at previous development stages and refed for 84 h on uninfected blood. Lane M, 100-bp DNA molecular mass; lane A, carcass of female adult; lane Ags, salivary glands of female adult, lane N, carcass of nymph; lane Ngs, salivary glands of nymph; lane T+, *B. bacilliformis* DNA; lane T–, nymph fed on uninfected ovine blood. B) *Bartonella* spp. DNA detection in blood isolated from feeders. Lane M, 100-bp DNA molecular mass marker; lane 48, ovine blood after 48 h of tick attachment on skin; lane 60, ovine blood after 60 h of tick attachment on skin; lane 72, ovine blood after 72 h of tick attachment on skin; lane 84, ovine blood after 84 h of tick attachment on skin; lane T+, *B. bacilliformis* DNA.

Eighteen nymphs and 13 female adults fed on *B*. *henselae*–infected blood at preceding life stages were refed for 84 h with uninfected blood. DNA extracts were prepared from SGs and carcasses of 7 partially engorged nymphs and 3 partially engorged females. *Bartonella* spp. DNA was amplified from carcasses and SGs of 4 nymphs and 1 female. For 1 nymph and 1 female, the specific 337-bp DNA fragment was amplified only in SG DNA extracts but not in carcass extracts (Table; Figure 3, panel **A**). Four pairs of SGs from 4 partially engorged females and 3 pairs of SGs from 3 partially engorged nymphs were incubated separately in Schneider *Drosphila* medium for 6 days before being placed on sheep blood agar. *B*. *henselae* colonies appeared after 7 days for all SGs tested, which indicated the presence of viable bacteria in SGs from adults and nymphs.

To determine whether *B*. *henselae* in SGs of nymphs and adults were infective, we infected 2 healthy cats with 1 pair of SGs from a nymph and 1 pair of SGs from a female adult. The cat infected with SGs from an adult became bacteremic 7 days after infection (5 × 10^5^ CFU/mL at day7, 6 × 10^6^ CFU/mL at day 14, and 1 × 10^6^ CFU/mL at day 21), and the cat infected with SGs from a nymph became bacteremic after 14 days of infection (8 × 10^5^ CFU/mL at day 14 and 2 × 10^7^ CFU/mL at day 21).

Blood samples from feeders were obtained every 12 h for the first 48 h to detect *B*. *henselae*. In the first 60 h, *B*. *henselae* DNA or viable *B*. *henselae* was not detected in blood samples. After 72 h of refeeding of infected ticks, *Bartonella* spp. DNA was successfully amplified (Figure 3, panel **B**), and *B*. *henselae* colonies were isolated from these blood samples after preincubation for 6 days in Schneider *Drosophila* medium, which indicated viability of the transmitted bacteria.

## Discussion

This study demonstrated transmission of *B*. *henselae* by *I*. *ricinus* ticks across different developmental stages, migration and multiplication of viable and infective *B*. *henselae* in SGs after a second blood meal, and transmission of *B*. *henselae* from ticks to blood. These findings indicate that *I*. *ricinus* is a competent vector for *B*. *henselae*.

Vector biologists and epidemiologists have suggested that ticks may play a role in transmission of *Bartonella* spp (*11*,*12*,*19*,*23*,*25*,*28*,*29*). This suggestion was based on indirect data for detection of bacterial DNA in ticks (*18*,*19*,*24*), humans exposed to tick bites (*30*), or serologic evidence of co-infection of humans with pathogens known to be transmitted by ticks (*11*,*36*). Difficulties in rearing *I*. *ricinus* and lack of a rodent model for *B*. *henselae* infection may explain the absence of data demonstrating the role of this tick as a vector of *B*. *henselae*. Recent development of an artificial method suitable for feeding ticks (*33*) enabled us to study experimental infection of ticks with blood containing *B*. *henselae*, to monitor *B*. *henselae* through various tick stages, and to evaluate putative transmission of bacteria from the tick to blood.

To select a tick population with the lowest *Bartonella* spp. DNA prevalence, we estimated the prevalence of *Bartonella* spp. DNA in questing *I*. *ricinus* collected in different areas in France. The lowest *Bartonella* spp. DNA prevalence was in Loire-Atlantique (*19* and unpub. data). We thus used ticks collected in this area for our study.

We detected *B*. *henselae* DNA in 100% of carcasses from nymphs and 67% of carcasses from adults fed on ovine blood containing *B*. *henselae* at their preceding stages. No *B*. *henselae* DNA was amplified in corresponding SGs in a nested PCR, which is more sensitive than amplification of the classic citrate synthase gene. This result demonstrated that bacteria could be ingested by *I*. *ricinus* larvae and nymphs during feeding on artificial skin and that bacterial DNA was maintained in the tick after molting. However, no or undetectable numbers reached the SGs.

Although bacterial DNA was detected in eggs laid by females fed on blood containing *B*. *henselae*, larvae obtained from these eggs were PCR-negative for *B. henselae*. This finding suggests external contamination of eggs with DNA rather than transovarial persistence of bacteria.

When molted nymphs and female ticks potentially contaminated with *B*. *henselae* at their previous developmental stage were refed on uninfected blood, viable *B*. *henselae* were detected in SGs after 84 h of engorgement. Two hypotheses could explain the absence of detectable bacterial DNA in SGs after an infected blood meal and molting, when it becomes detectable after a partial refeeding blood meal. The first hypothesis is that the 84-h refeeding period may act as a stimulus and enable migration of bacteria from the gut to SGs of the tick, as previously described for *B*. *burgdorferi* sensu lato (*14*). The second hypothesis is that this refeeding period may stimulate multiplication of bacteria already present in SGs, but at undetectable levels. More investigations are needed to validate one of these hypotheses. Bacteria located in SGs of nymphs and adults are infective because injection of 1 pair of infected SGs into cats induced high levels of bacteremia. Cats became bacteremic in the first 2 weeks after injection, as described for cat infection with *B*. *henselae* by fleas, and bacteremia levels were similar to those observed in cats infected by flea bites (*7*,*8*).

Viable *B*. *henselae* in blood after 72 h of feeding of ticks with *B*. *henselae*–infected ticks demonstrated its transmission from the tick to the blood by mouthparts of the ticks. The duration of multiplication or migration described above would explain such a delay in bacterial transmission.

Because our results have demonstrated competence of *I*. *ricinus* for transmission of *B*. *henselae*, cat models of *B*. *henselae* transmission by ticks are needed to confirm that cats can be infected with *B*. *henselae* by tick bites. Further investigations are also needed to evaluate the capacity of *I*. *ricinus* to transmit *B*. *henselae* to cats and humans. Such transmission could occur because cats, although not common hosts for *I*. *ricinus*, can be infested with this tick. In France, attached *I*. *ricinus* are commonly found on cats brought to veterinarians (J. Guillot, pers. comm.). In Great Britain, Ogden et al. (*37*) reported cats with woodland and moorland habitats as hosts for *I*. *ricinus*. Podsiadly et al. (*38*) reported *B*. *henselae* in cats and in *I*. *ricinus* removed from those cats in Poland.

In conclusion, we demonstrated by using feeding on artificial skin that *B*. *henselae*, the cause of cat-scratch disease in humans, could be transmitted by ticks through saliva. Although further investigations are needed to clarify the epidemiology of such transmission, health authorities must take into account the possibility of bartonellosis in persons exposed to tick bites, and *B*. *henselae* must be identified as a tick-borne pathogen.
